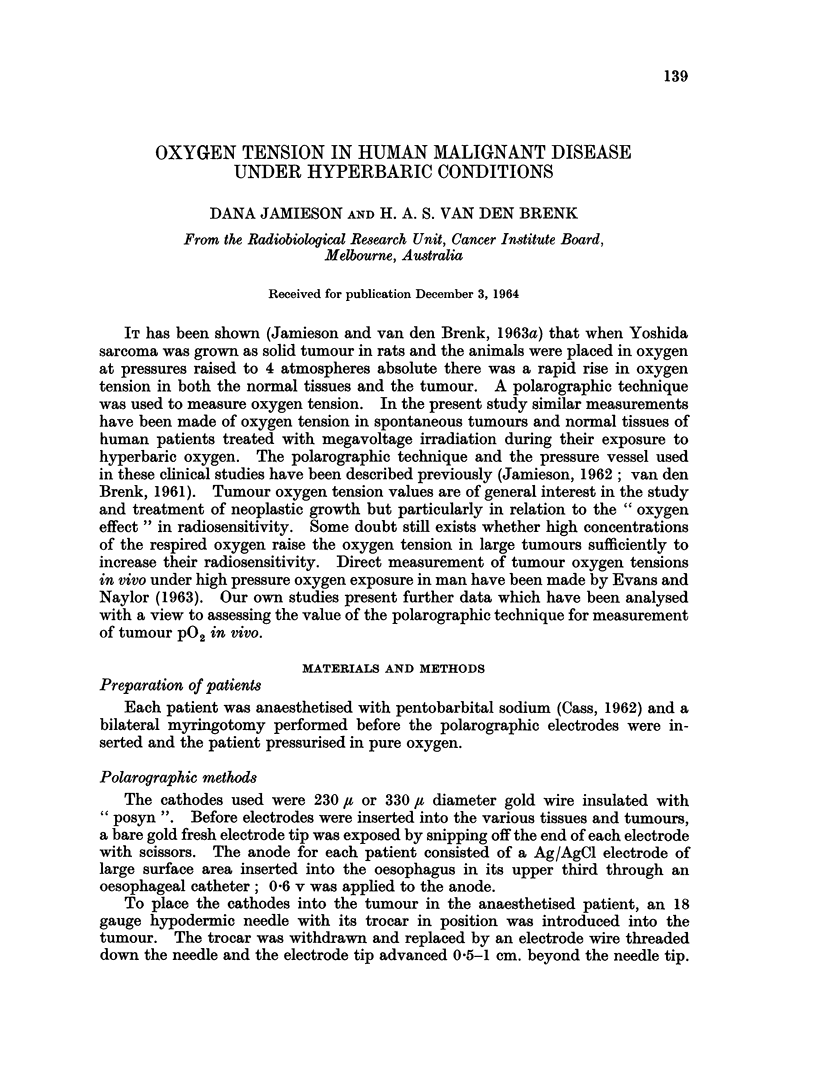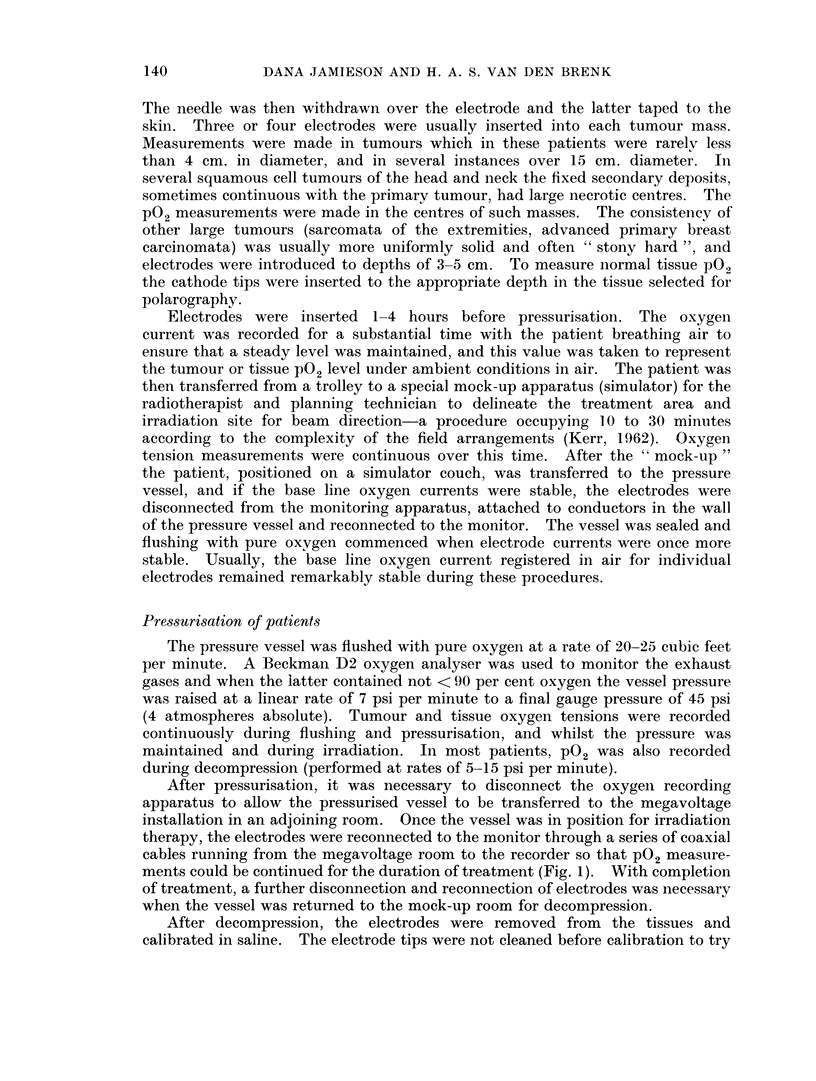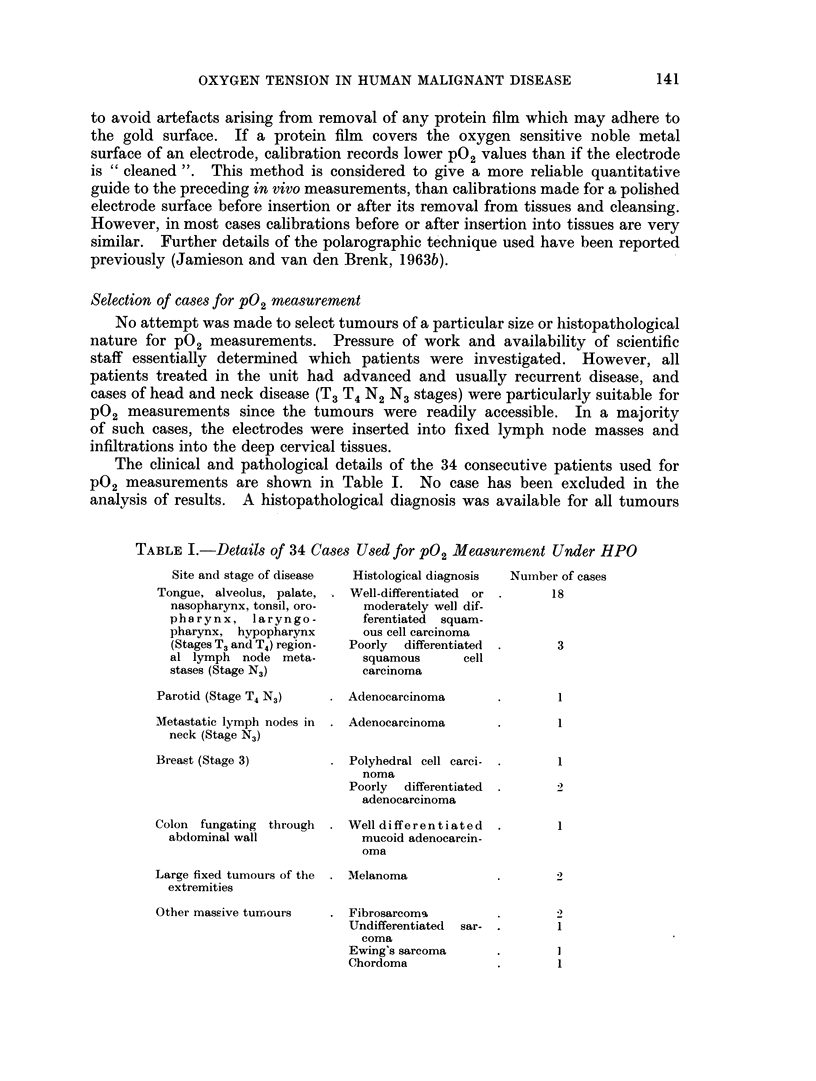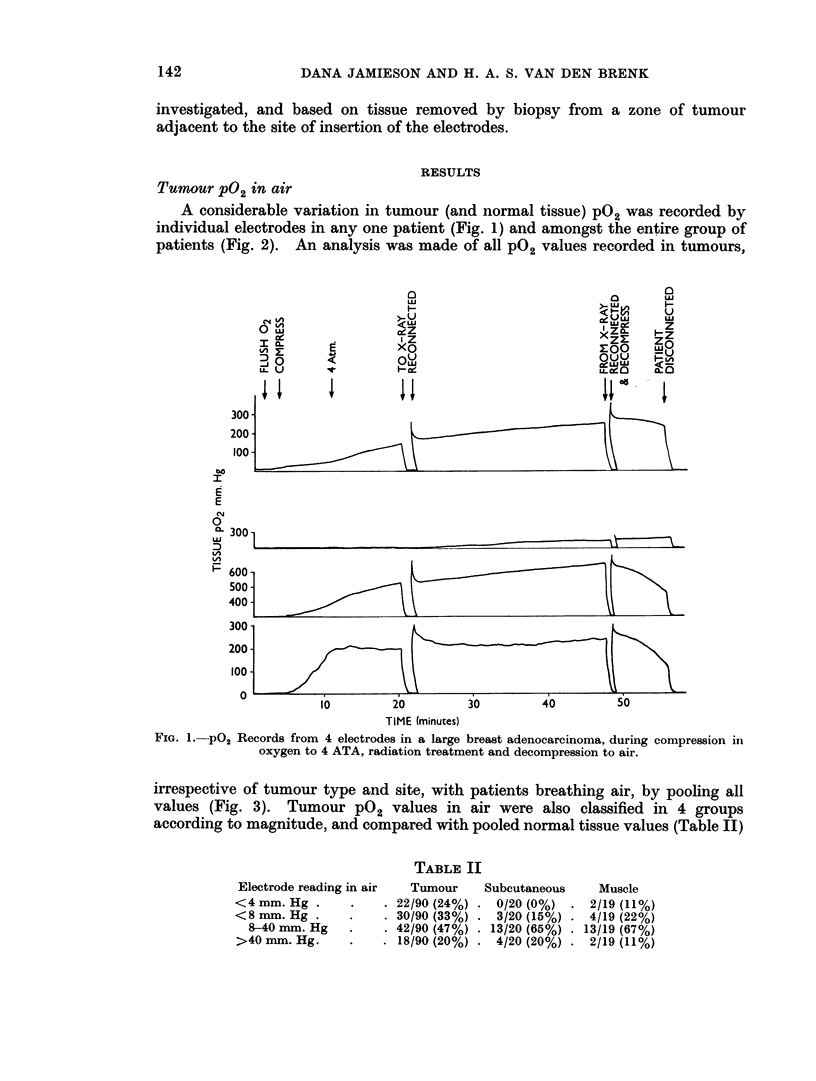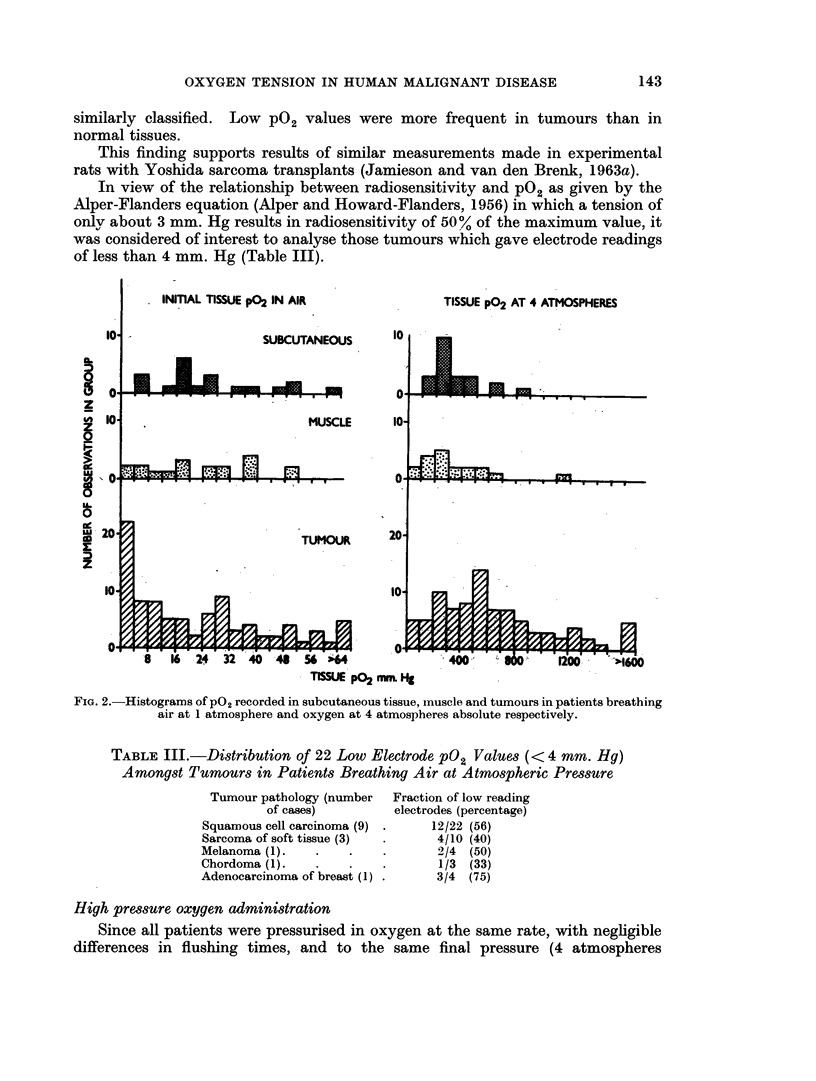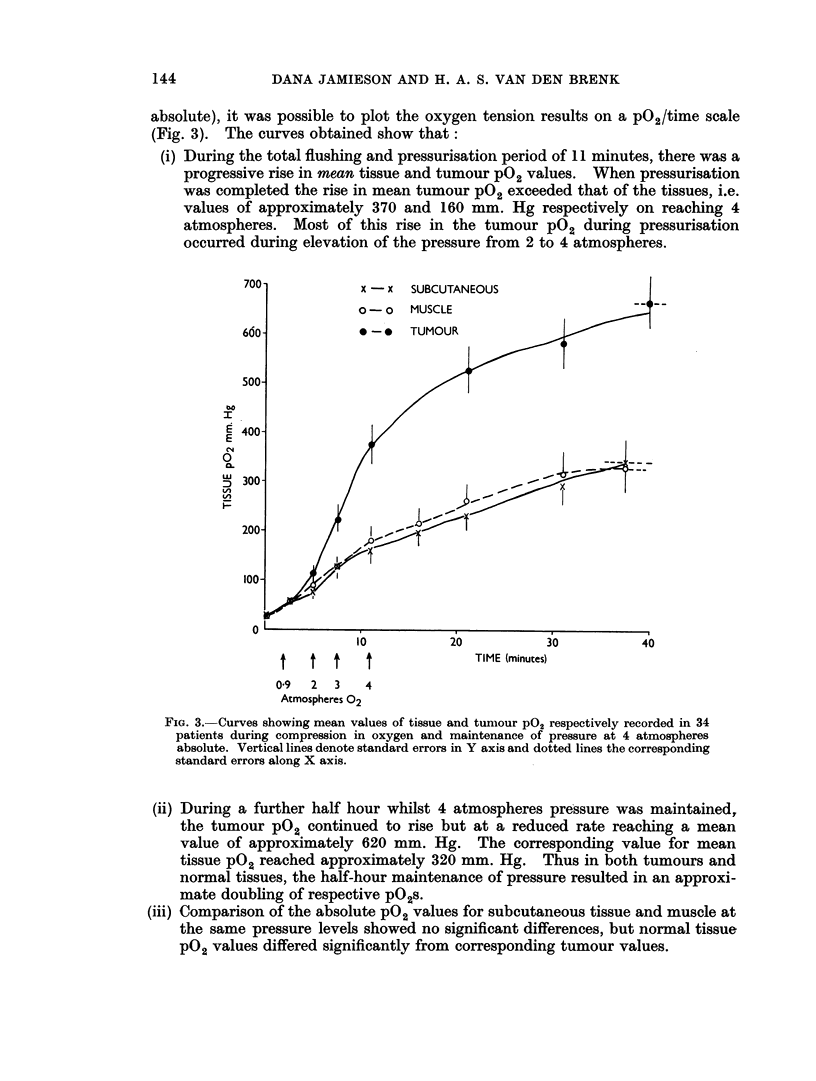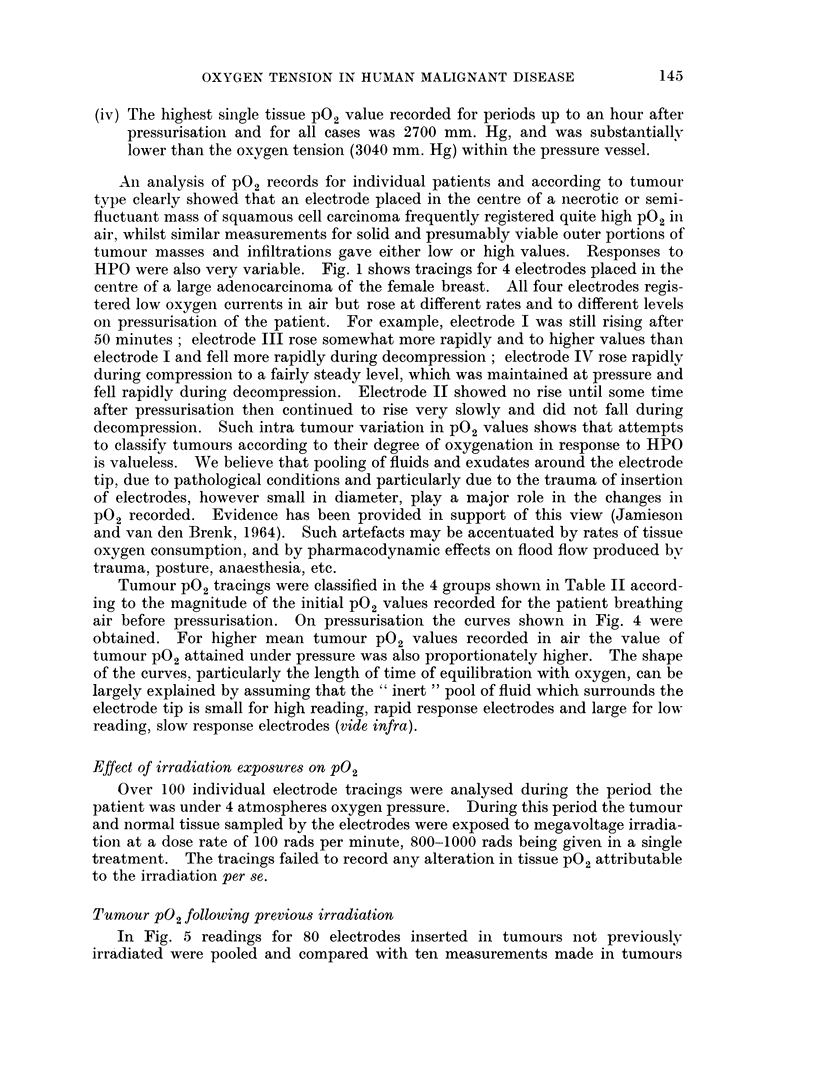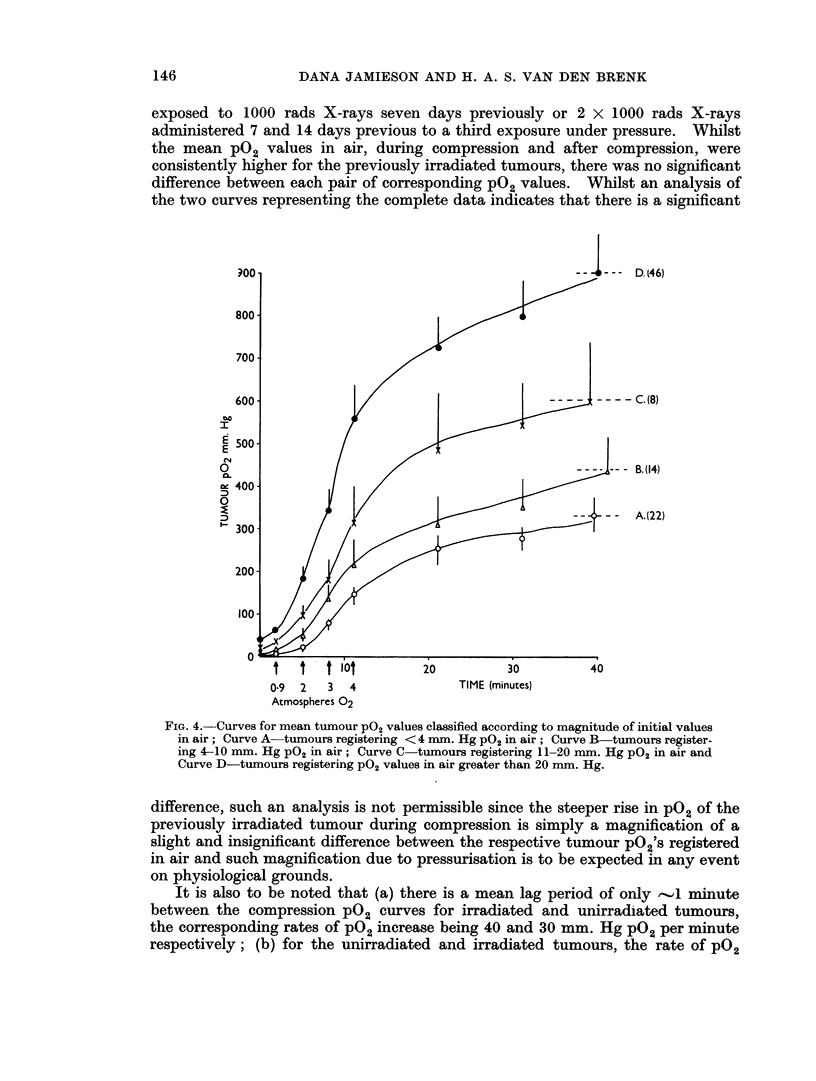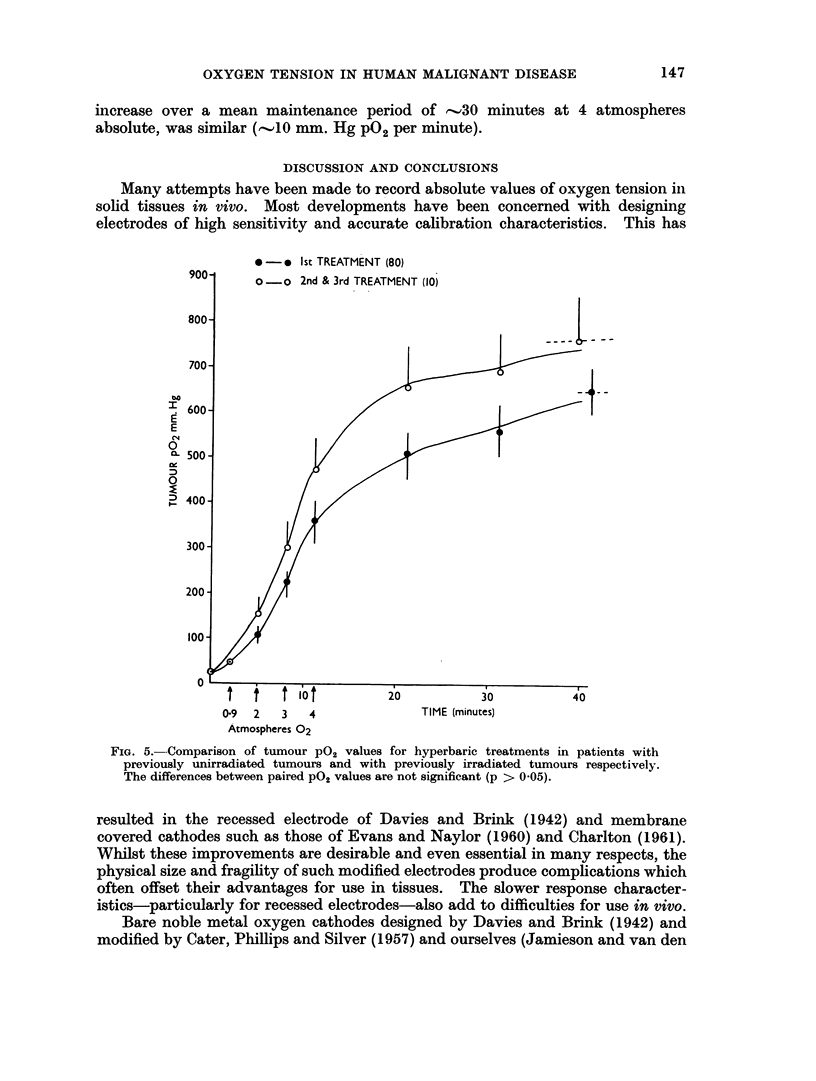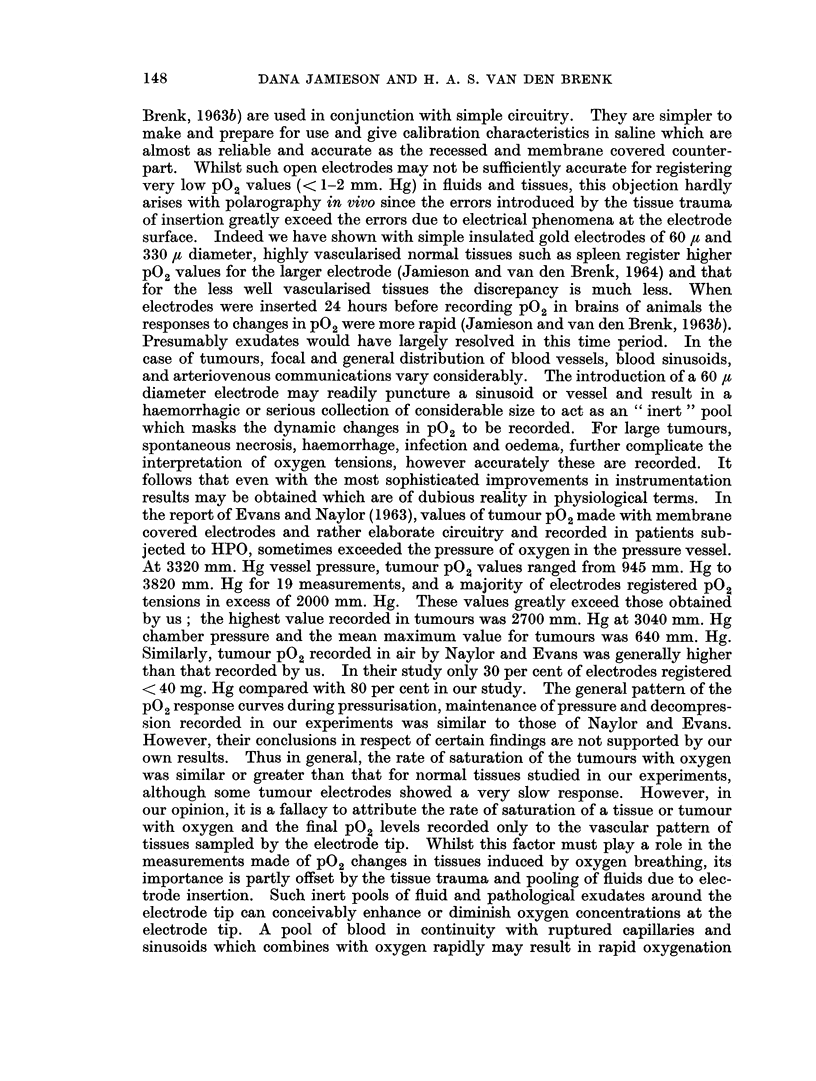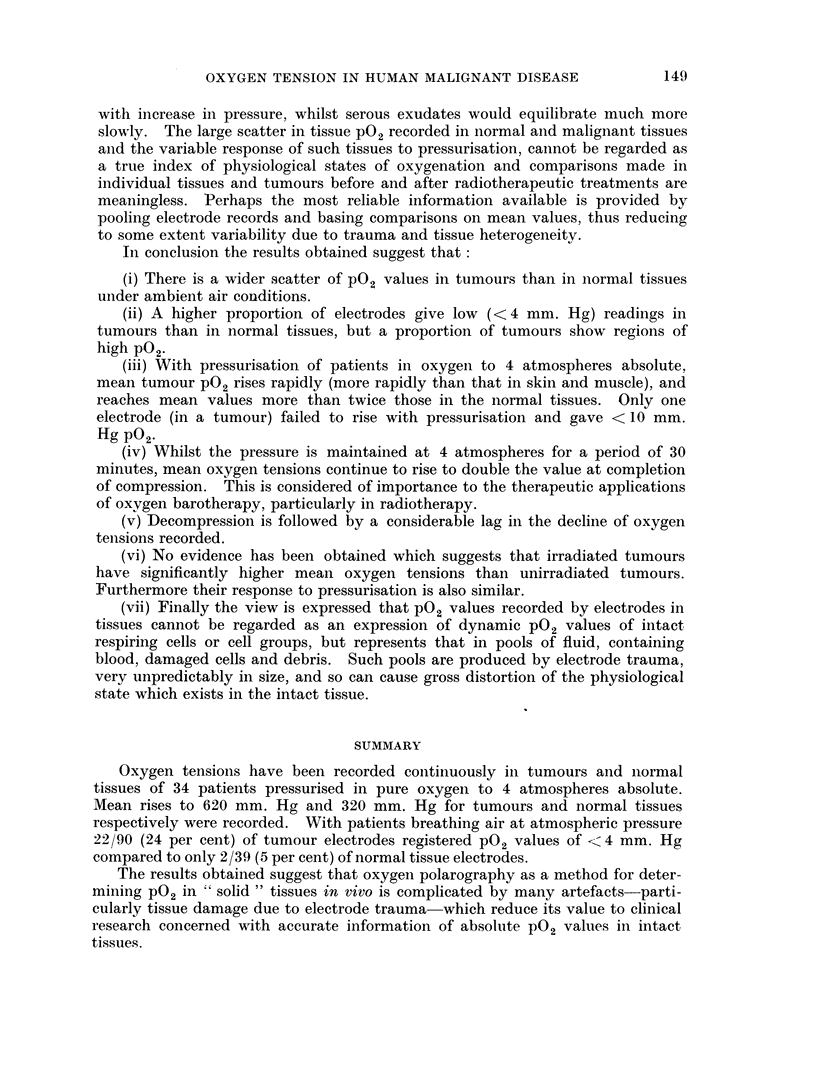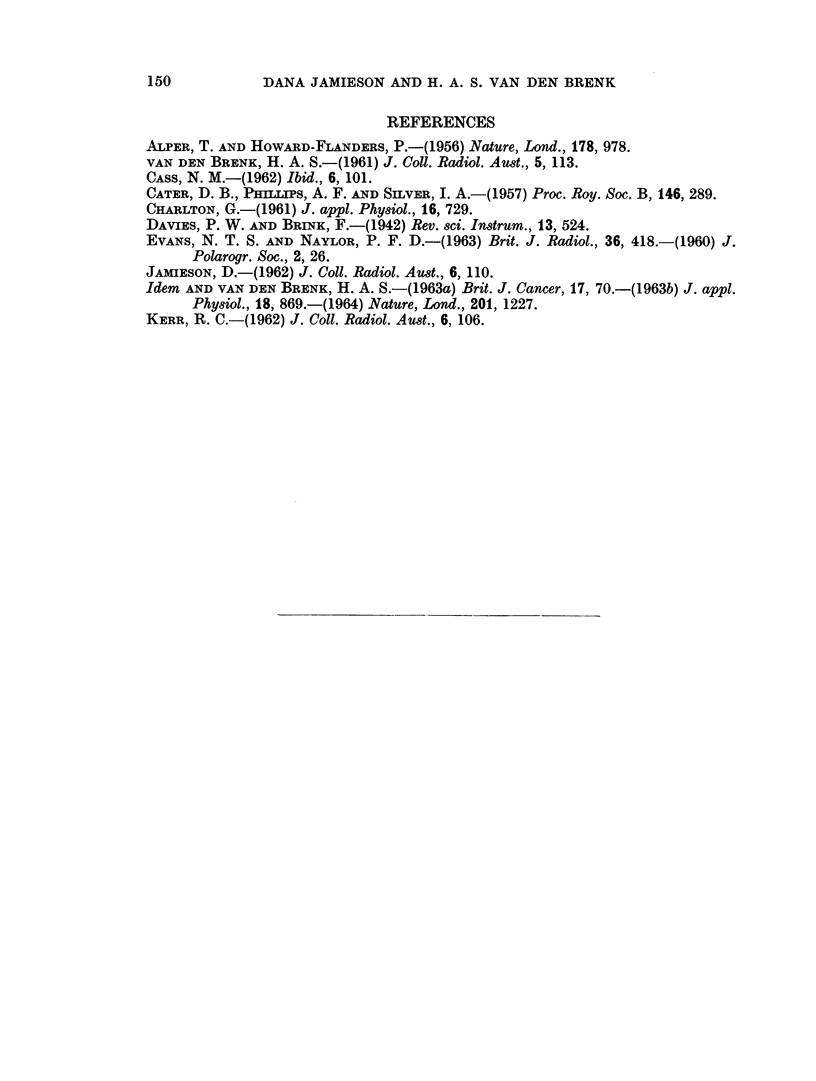# Oxygen Tension in Human Malignant Disease under Hyperbaric Conditions

**DOI:** 10.1038/bjc.1965.16

**Published:** 1965-03

**Authors:** Dana Jamieson, H. A. S. van den Brenk


### OXYGEN TENSION IN HUMAN MALIGNANT DISEASE UNDER HYPERBARIC CONDITIONS

DANA JAMIESON AND H. A. S. VAN DEN BRENK

*From the Radiobiological Research Unit, Cancer Institute Board, Melbourne, Australia*

Received for publication December 3, 1964

IT has been shown (Jamieson and van den Brenk, 1963*a*) that when Yoshida sarcoma was grown as solid tumour in rats and the animals were placed in oxygen at pressures raised to 4 atmospheres absolute there was a rapid rise in oxygen tension in both the normal tissues and the tumour. A polarographic technique was used to measure oxygen tension. In the present study similar measurements have been made of oxygen tension in spontaneous tumours and normal tissues of human patients treated with megavoltage irradiation during their exposure to hyperbaric oxygen. The polarographic technique and the pressure vessel used in these clinical studies have been described previously (Jamieson, 1962 ; van den Brenk, 1961). Tumour oxygen tension values are of general interest in the study and treatment of neoplastic growth but particularly in relation to the " oxygen effect " in radiosensitivity. Some doubt still exists whether high concentrations of the respired oxygen raise the oxygen tension in large tumours sufficiently to increase their radiosensitivity. Direct measurement of tumour oxygen tensions *in vivo* under high pressure oxygen exposure in man have been made by Evans and Naylor (1963). Our own studies present further data which have been analysed with a view to assessing the value of the polarographic technique for measurement of tumour $pO_2$ *in vivo.*

### MATERIALS AND METHODS

### *Preparation of patients*

Each patient was anaesthetised with pentobarbital sodium (Cass, 1962) and a bilateral myringotomy performed before the polarographic electrodes were inserted and the patient pressurised in pure oxygen.

### *Polarographic methods*

The cathodes used were 230 $\mu$ or 330 $\mu$ diameter gold wire insulated with " posyn ". Before electrodes were inserted into the various tissues and tumours, a bare gold fresh electrode tip was exposed by snipping off the end of each electrode with scissors. The anode for each patient consisted of a Ag/AgCl electrode of large surface area inserted into the oesophagus in its upper third through an oesophageal catheter ; 0·6 v was applied to the anode.

To place the cathodes into the tumour in the anaesthetised patient, an 18 gauge hypodermic needle with its trocar in position was introduced into the tumour. The trocar was withdrawn and replaced by an electrode wire threaded down the needle and the electrode tip advanced 0·5–1 cm. beyond the needle tip.

The needle was then withdrawn over the electrode and the latter taped to the skin. Three or four electrodes were usually inserted into each tumour mass. Measurements were made in tumours which in these patients were rarely less than 4 cm. in diameter, and in several instances over 15 cm. diameter. In several squamous cell tumours of the head and neck the fixed secondary deposits, sometimes continuous with the primary tumour, had large necrotic centres. The $pO_2$ measurements were made in the centres of such masses. The consistency of other large tumours (sarcomata of the extremities, advanced primary breast carcinomata) was usually more uniformly solid and often " stony hard ", and electrodes were introduced to depths of 3–5 cm. To measure normal tissue $pO_2$ the cathode tips were inserted to the appropriate depth in the tissue selected for polarography.

Electrodes were inserted 1–4 hours before pressurisation. The oxygen current was recorded for a substantial time with the patient breathing air to ensure that a steady level was maintained, and this value was taken to represent the tumour or tissue $pO_2$ level under ambient conditions in air. The patient was then transferred from a trolley to a special mock-up apparatus (simulator) for the radiotherapist and planning technician to delineate the treatment area and irradiation site for beam direction—a procedure occupying 10 to 30 minutes according to the complexity of the field arrangements (Kerr, 1962). Oxygen tension measurements were continuous over this time. After the " mock-up " the patient, positioned on a simulator couch, was transferred to the pressure vessel, and if the base line oxygen currents were stable, the electrodes were disconnected from the monitoring apparatus, attached to conductors in the wall of the pressure vessel and reconnected to the monitor. The vessel was sealed and flushing with pure oxygen commenced when electrode currents were once more stable. Usually, the base line oxygen current registered in air for individual electrodes remained remarkably stable during these procedures.

*Pressurisation of patients*

The pressure vessel was flushed with pure oxygen at a rate of 20–25 cubic feet per minute. A Beckman D2 oxygen analyser was used to monitor the exhaust gases and when the latter contained not $<90$ per cent oxygen the vessel pressure was raised at a linear rate of 7 psi per minute to a final gauge pressure of 45 psi (4 atmospheres absolute). Tumour and tissue oxygen tensions were recorded continuously during flushing and pressurisation, and whilst the pressure was maintained and during irradiation. In most patients, $pO_2$ was also recorded during decompression (performed at rates of 5–15 psi per minute).

After pressurisation, it was necessary to disconnect the oxygen recording apparatus to allow the pressurised vessel to be transferred to the megavoltage installation in an adjoining room. Once the vessel was in position for irradiation therapy, the electrodes were reconnected to the monitor through a series of coaxial cables running from the megavoltage room to the recorder so that $pO_2$ measurements could be continued for the duration of treatment (Fig. 1). With completion of treatment, a further disconnection and reconnection of electrodes was necessary when the vessel was returned to the mock-up room for decompression.

After decompression, the electrodes were removed from the tissues and calibrated in saline. The electrode tips were not cleaned before calibration to try to avoid artefacts arising from removal of any protein film which may adhere to the gold surface. If a protein film covers the oxygen sensitive noble metal surface of an electrode, calibration records lower $pO_2$ values than if the electrode is "cleaned". This method is considered to give a more reliable quantitative guide to the preceding *in vivo* measurements, than calibrations made for a polished electrode surface before insertion or after its removal from tissues and cleansing. However, in most cases calibrations before or after insertion into tissues are very similar. Further details of the polarographic technique used have been reported previously (Jamieson and van den Brenk, 1963*b*).

*Selection of cases for $pO_2$ measurement*

No attempt was made to select tumours of a particular size or histopathological nature for $pO_2$ measurements. Pressure of work and availability of scientific staff essentially determined which patients were investigated. However, all patients treated in the unit had advanced and usually recurrent disease, and cases of head and neck disease ($T_3$ $T_4$ $N_2$ $N_3$ stages) were particularly suitable for $pO_2$ measurements since the tumours were readily accessible. In a majority of such cases, the electrodes were inserted into fixed lymph node masses and infiltrations into the deep cervical tissues.

The clinical and pathological details of the 34 consecutive patients used for $pO_2$ measurements are shown in Table I. No case has been excluded in the analysis of results. A histopathological diagnosis was available for all tumours

TABLE I.—*Details of* 34 *Cases Used for $pO_2$ Measurement Under HPO*

| Site and stage of disease | Histological diagnosis | Number of cases |
|---|---|---|
| Tongue, alveolus, palate, nasopharynx, tonsil, oropharynx, laryngopharynx, hypopharynx (Stages $T_3$ and $T_4$) regional lymph node metastases (Stage $N_3$) | Well-differentiated or moderately well differentiated squamous cell carcinoma | 18 |
| | Poorly differentiated squamous cell carcinoma | 3 |
| Parotid (Stage $T_4$ $N_3$) | Adenocarcinoma | 1 |
| Metastatic lymph nodes in neck (Stage $N_3$) | Adenocarcinoma | 1 |
| Breast (Stage 3) | Polyhedral cell carcinoma | 1 |
| | Poorly differentiated adenocarcinoma | 2 |
| Colon fungating through abdominal wall | Well differentiated mucoid adenocarcinoma | 1 |
| Large fixed tumours of the extremities | Melanoma | 2 |
| Other massive tumours | Fibrosarcoma | 2 |
| | Undifferentiated sarcoma | 1 |
| | Ewing's sarcoma | 1 |
| | Chordoma | 1 |

investigated, and based on tissue removed by biopsy from a zone of tumour adjacent to the site of insertion of the electrodes.

### RESULTS

### *Tumour $pO_2$ in air*

A considerable variation in tumour (and normal tissue) $pO_2$ was recorded by individual electrodes in any one patient (Fig. 1) and amongst the entire group of patients (Fig. 2). An analysis was made of all $pO_2$ values recorded in tumours,

FIG. 1.—$pO_2$ Records from 4 electrodes in a large breast adenocarcinoma, during compression in oxygen to 4 ATA, radiation treatment and decompression to air.

irrespective of tumour type and site, with patients breathing air, by pooling all values (Fig. 3). Tumour $pO_2$ values in air were also classified in 4 groups according to magnitude, and compared with pooled normal tissue values (Table II)

TABLE II

| Electrode reading in air | Tumour | Subcutaneous | Muscle |
|---|---|---|---|
| <4 mm. Hg | 22/90 (24%) | 0/20 (0%) | 2/19 (11%) |
| <8 mm. Hg | 30/90 (33%) | 3/20 (15%) | 4/19 (22%) |
| 8–40 mm. Hg | 42/90 (47%) | 13/20 (65%) | 13/19 (67%) |
| >40 mm. Hg | 18/90 (20%) | 4/20 (20%) | 2/19 (11%) |

similarly classified. Low $pO_2$ values were more frequent in tumours than in normal tissues.

This finding supports results of similar measurements made in experimental rats with Yoshida sarcoma transplants (Jamieson and van den Brenk, 1963*a*).

In view of the relationship between radiosensitivity and $pO_2$ as given by the Alper-Flanders equation (Alper and Howard-Flanders, 1956) in which a tension of only about 3 mm. Hg results in radiosensitivity of 50% of the maximum value, it was considered of interest to analyse those tumours which gave electrode readings of less than 4 mm. Hg (Table III).

FIG. 2.—Histograms of $pO_2$ recorded in subcutaneous tissue, muscle and tumours in patients breathing air at 1 atmosphere and oxygen at 4 atmospheres absolute respectively.

TABLE III.—*Distribution of 22 Low Electrode $pO_2$ Values ($<$ 4 mm. Hg) Amongst Tumours in Patients Breathing Air at Atmospheric Pressure*

| Tumour pathology (number of cases) | Fraction of low reading electrodes (percentage) |
|---|---|
| Squamous cell carcinoma (9) . | 12/22 (56) |
| Sarcoma of soft tissue (3) . | 4/10 (40) |
| Melanoma (1). . . . | 2/4 (50) |
| Chordoma (1). . . . | 1/3 (33) |
| Adenocarcinoma of breast (1) . | 3/4 (75) |

### *High pressure oxygen administration*

Since all patients were pressurised in oxygen at the same rate, with negligible differences in flushing times, and to the same final pressure (4 atmospheres absolute), it was possible to plot the oxygen tension results on a $pO_2$/time scale (Fig. 3). The curves obtained show that :

(i) During the total flushing and pressurisation period of 11 minutes, there was a progressive rise in *mean* tissue and tumour $pO_2$ values. When pressurisation was completed the rise in mean tumour $pO_2$ exceeded that of the tissues, i.e. values of approximately 370 and 160 mm. Hg respectively on reaching 4 atmospheres. Most of this rise in the tumour $pO_2$ during pressurisation occurred during elevation of the pressure from 2 to 4 atmospheres.

FIG. 3.—Curves showing mean values of tissue and tumour $pO_2$ respectively recorded in 34 patients during compression in oxygen and maintenance of pressure at 4 atmospheres absolute. Vertical lines denote standard errors in Y axis and dotted lines the corresponding standard errors along X axis.

(ii) During a further half hour whilst 4 atmospheres pressure was maintained, the tumour $pO_2$ continued to rise but at a reduced rate reaching a mean value of approximately 620 mm. Hg. The corresponding value for mean tissue $pO_2$ reached approximately 320 mm. Hg. Thus in both tumours and normal tissues, the half-hour maintenance of pressure resulted in an approximate doubling of respective $pO_2$s.

(iii) Comparison of the absolute $pO_2$ values for subcutaneous tissue and muscle at the same pressure levels showed no significant differences, but normal tissue $pO_2$ values differed significantly from corresponding tumour values.

(iv) The highest single tissue $pO_2$ value recorded for periods up to an hour after pressurisation and for all cases was 2700 mm. Hg, and was substantially lower than the oxygen tension (3040 mm. Hg) within the pressure vessel.

An analysis of $pO_2$ records for individual patients and according to tumour type clearly showed that an electrode placed in the centre of a necrotic or semi-fluctuant mass of squamous cell carcinoma frequently registered quite high $pO_2$ in air, whilst similar measurements for solid and presumably viable outer portions of tumour masses and infiltrations gave either low or high values. Responses to HPO were also very variable. Fig. 1 shows tracings for 4 electrodes placed in the centre of a large adenocarcinoma of the female breast. All four electrodes registered low oxygen currents in air but rose at different rates and to different levels on pressurisation of the patient. For example, electrode I was still rising after 50 minutes ; electrode III rose somewhat more rapidly and to higher values than electrode I and fell more rapidly during decompression ; electrode IV rose rapidly during compression to a fairly steady level, which was maintained at pressure and fell rapidly during decompression. Electrode II showed no rise until some time after pressurisation then continued to rise very slowly and did not fall during decompression. Such intra tumour variation in $pO_2$ values shows that attempts to classify tumours according to their degree of oxygenation in response to HPO is valueless. We believe that pooling of fluids and exudates around the electrode tip, due to pathological conditions and particularly due to the trauma of insertion of electrodes, however small in diameter, play a major role in the changes in $pO_2$ recorded. Evidence has been provided in support of this view (Jamieson and van den Brenk, 1964). Such artefacts may be accentuated by rates of tissue oxygen consumption, and by pharmacodynamic effects on flood flow produced by trauma, posture, anaesthesia, etc.

Tumour $pO_2$ tracings were classified in the 4 groups shown in Table II according to the magnitude of the initial $pO_2$ values recorded for the patient breathing air before pressurisation. On pressurisation the curves shown in Fig. 4 were obtained. For higher mean tumour $pO_2$ values recorded in air the value of tumour $pO_2$ attained under pressure was also proportionately higher. The shape of the curves, particularly the length of time of equilibration with oxygen, can be largely explained by assuming that the " inert " pool of fluid which surrounds the electrode tip is small for high reading, rapid response electrodes and large for low reading, slow response electrodes (*vide infra*).

*Effect of irradiation exposures on $pO_2$*

Over 100 individual electrode tracings were analysed during the period the patient was under 4 atmospheres oxygen pressure. During this period the tumour and normal tissue sampled by the electrodes were exposed to megavoltage irradiation at a dose rate of 100 rads per minute, 800–1000 rads being given in a single treatment. The tracings failed to record any alteration in tissue $pO_2$ attributable to the irradiation *per se*.

*Tumour $pO_2$ following previous irradiation*

In Fig. 5 readings for 80 electrodes inserted in tumours not previously irradiated were pooled and compared with ten measurements made in tumours exposed to 1000 rads X-rays seven days previously or 2 × 1000 rads X-rays administered 7 and 14 days previous to a third exposure under pressure. Whilst the mean $pO_2$ values in air, during compression and after compression, were consistently higher for the previously irradiated tumours, there was no significant difference between each pair of corresponding $pO_2$ values. Whilst an analysis of the two curves representing the complete data indicates that there is a significant

FIG. 4.—Curves for mean tumour $pO_2$ values classified according to magnitude of initial values in air ; Curve A—tumours registering $<4$ mm. Hg $pO_2$ in air ; Curve B—tumours registering 4–10 mm. Hg $pO_2$ in air ; Curve C—tumours registering 11–20 mm. Hg $pO_2$ in air and Curve D—tumours registering $pO_2$ values in air greater than 20 mm. Hg.

difference, such an analysis is not permissible since the steeper rise in $pO_2$ of the previously irradiated tumour during compression is simply a magnification of a slight and insignificant difference between the respective tumour $pO_2$'s registered in air and such magnification due to pressurisation is to be expected in any event on physiological grounds.

It is also to be noted that (a) there is a mean lag period of only $\sim$1 minute between the compression $pO_2$ curves for irradiated and unirradiated tumours, the corresponding rates of $pO_2$ increase being 40 and 30 mm. Hg $pO_2$ per minute respectively ; (b) for the unirradiated and irradiated tumours, the rate of $pO_2$

increase over a mean maintenance period of ~30 minutes at 4 atmospheres absolute, was similar (~10 mm. Hg $pO_2$ per minute).

### DISCUSSION AND CONCLUSIONS

Many attempts have been made to record absolute values of oxygen tension in solid tissues *in vivo*. Most developments have been concerned with designing electrodes of high sensitivity and accurate calibration characteristics. This has

FIG. 5.—Comparison of tumour $pO_2$ values for hyperbaric treatments in patients with previously unirradiated tumours and with previously irradiated tumours respectively. The differences between paired $pO_2$ values are not significant ($p > 0{\cdot}05$).

resulted in the recessed electrode of Davies and Brink (1942) and membrane covered cathodes such as those of Evans and Naylor (1960) and Charlton (1961). Whilst these improvements are desirable and even essential in many respects, the physical size and fragility of such modified electrodes produce complications which often offset their advantages for use in tissues. The slower response characteristics—particularly for recessed electrodes—also add to difficulties for use *in vivo*.

Bare noble metal oxygen cathodes designed by Davies and Brink (1942) and modified by Cater, Phillips and Silver (1957) and ourselves (Jamieson and van den

Brenk, 1963*b*) are used in conjunction with simple circuitry. They are simpler to make and prepare for use and give calibration characteristics in saline which are almost as reliable and accurate as the recessed and membrane covered counterpart. Whilst such open electrodes may not be sufficiently accurate for registering very low $pO_2$ values (< 1–2 mm. Hg) in fluids and tissues, this objection hardly arises with polarography *in vivo* since the errors introduced by the tissue trauma of insertion greatly exceed the errors due to electrical phenomena at the electrode surface. Indeed we have shown with simple insulated gold electrodes of 60 $\mu$ and 330 $\mu$ diameter, highly vascularised normal tissues such as spleen register higher $pO_2$ values for the larger electrode (Jamieson and van den Brenk, 1964) and that for the less well vascularised tissues the discrepancy is much less. When electrodes were inserted 24 hours before recording $pO_2$ in brains of animals the responses to changes in $pO_2$ were more rapid (Jamieson and van den Brenk, 1963*b*). Presumably exudates would have largely resolved in this time period. In the case of tumours, focal and general distribution of blood vessels, blood sinusoids, and arteriovenous communications vary considerably. The introduction of a 60 $\mu$ diameter electrode may readily puncture a sinusoid or vessel and result in a haemorrhagic or serious collection of considerable size to act as an " inert " pool which masks the dynamic changes in $pO_2$ to be recorded. For large tumours, spontaneous necrosis, haemorrhage, infection and oedema, further complicate the interpretation of oxygen tensions, however accurately these are recorded. It follows that even with the most sophisticated improvements in instrumentation results may be obtained which are of dubious reality in physiological terms. In the report of Evans and Naylor (1963), values of tumour $pO_2$ made with membrane covered electrodes and rather elaborate circuitry and recorded in patients subjected to HPO, sometimes exceeded the pressure of oxygen in the pressure vessel. At 3320 mm. Hg vessel pressure, tumour $pO_2$ values ranged from 945 mm. Hg to 3820 mm. Hg for 19 measurements, and a majority of electrodes registered $pO_2$ tensions in excess of 2000 mm. Hg. These values greatly exceed those obtained by us ; the highest value recorded in tumours was 2700 mm. Hg at 3040 mm. Hg chamber pressure and the mean maximum value for tumours was 640 mm. Hg. Similarly, tumour $pO_2$ recorded in air by Naylor and Evans was generally higher than that recorded by us. In their study only 30 per cent of electrodes registered < 40 mg. Hg compared with 80 per cent in our study. The general pattern of the $pO_2$ response curves during pressurisation, maintenance of pressure and decompression recorded in our experiments was similar to those of Naylor and Evans. However, their conclusions in respect of certain findings are not supported by our own results. Thus in general, the rate of saturation of the tumours with oxygen was similar or greater than that for normal tissues studied in our experiments, although some tumour electrodes showed a very slow response. However, in our opinion, it is a fallacy to attribute the rate of saturation of a tissue or tumour with oxygen and the final $pO_2$ levels recorded only to the vascular pattern of tissues sampled by the electrode tip. Whilst this factor must play a role in the measurements made of $pO_2$ changes in tissues induced by oxygen breathing, its importance is partly offset by the tissue trauma and pooling of fluids due to electrode insertion. Such inert pools of fluid and pathological exudates around the electrode tip can conceivably enhance or diminish oxygen concentrations at the electrode tip. A pool of blood in continuity with ruptured capillaries and sinusoids which combines with oxygen rapidly may result in rapid oxygenation with increase in pressure, whilst serous exudates would equilibrate much more slowly. The large scatter in tissue $pO_2$ recorded in normal and malignant tissues and the variable response of such tissues to pressurisation, cannot be regarded as a true index of physiological states of oxygenation and comparisons made in individual tissues and tumours before and after radiotherapeutic treatments are meaningless. Perhaps the most reliable information available is provided by pooling electrode records and basing comparisons on mean values, thus reducing to some extent variability due to trauma and tissue heterogeneity.

In conclusion the results obtained suggest that :

(i) There is a wider scatter of $pO_2$ values in tumours than in normal tissues under ambient air conditions.

(ii) A higher proportion of electrodes give low ($<4$ mm. Hg) readings in tumours than in normal tissues, but a proportion of tumours show regions of high $pO_2$.

(iii) With pressurisation of patients in oxygen to 4 atmospheres absolute, mean tumour $pO_2$ rises rapidly (more rapidly than that in skin and muscle), and reaches mean values more than twice those in the normal tissues. Only one electrode (in a tumour) failed to rise with pressurisation and gave $<10$ mm. Hg $pO_2$.

(iv) Whilst the pressure is maintained at 4 atmospheres for a period of 30 minutes, mean oxygen tensions continue to rise to double the value at completion of compression. This is considered of importance to the therapeutic applications of oxygen barotherapy, particularly in radiotherapy.

(v) Decompression is followed by a considerable lag in the decline of oxygen tensions recorded.

(vi) No evidence has been obtained which suggests that irradiated tumours have significantly higher mean oxygen tensions than unirradiated tumours. Furthermore their response to pressurisation is also similar.

(vii) Finally the view is expressed that $pO_2$ values recorded by electrodes in tissues cannot be regarded as an expression of dynamic $pO_2$ values of intact respiring cells or cell groups, but represents that in pools of fluid, containing blood, damaged cells and debris. Such pools are produced by electrode trauma, very unpredictably in size, and so can cause gross distortion of the physiological state which exists in the intact tissue.

### SUMMARY

Oxygen tensions have been recorded continuously in tumours and normal tissues of 34 patients pressurised in pure oxygen to 4 atmospheres absolute. Mean rises to 620 mm. Hg and 320 mm. Hg for tumours and normal tissues respectively were recorded. With patients breathing air at atmospheric pressure 22/90 (24 per cent) of tumour electrodes registered $pO_2$ values of $<4$ mm. Hg compared to only 2/39 (5 per cent) of normal tissue electrodes.

The results obtained suggest that oxygen polarography as a method for determining $pO_2$ in " solid " tissues *in vivo* is complicated by many artefacts—particularly tissue damage due to electrode trauma—which reduce its value to clinical research concerned with accurate information of absolute $pO_2$ values in intact tissues.

### REFERENCES

ALPER, T. AND HOWARD-FLANDERS, P.—(1956) *Nature, Lond.*, **178**, 978.
VAN DEN BRENK, H. A. S.—(1961) *J. Coll. Radiol. Aust.*, **5**, 113.
CASS, N. M.—(1962) *Ibid.*, **6**, 101.
CATER, D. B., PHILLIPS, A. F. AND SILVER, I. A.—(1957) *Proc. Roy. Soc.* B, **146**, 289.
CHARLTON, G.—(1961) *J. appl. Physiol.*, **16**, 729.
DAVIES, P. W. AND BRINK, F.—(1942) *Rev. sci. Instrum.*, **13**, 524.
EVANS, N. T. S. AND NAYLOR, P. F. D.—(1963) *Brit. J. Radiol.*, **36**, 418.—(1960) *J. Polarogr. Soc.*, **2**, 26.
JAMIESON, D.—(1962) *J. Coll. Radiol. Aust.*, **6**, 110.
*Idem* AND VAN DEN BRENK, H. A. S.—(1963*a*) *Brit. J. Cancer*, **17**, 70.—(1963*b*) *J. appl. Physiol.*, **18**, 869.—(1964) *Nature, Lond.*, **201**, 1227.
KERR, R. C.—(1962) *J. Coll. Radiol. Aust.*, **6**, 106.